# Health behavior and disease self-management indicators in patients with cardiovascular diseases using a health app: Findings from an RCT

**DOI:** 10.3934/publichealth.2025015

**Published:** 2025-02-26

**Authors:** Sonia Lippke, Luisa Korte, Vinayak Anand Kumar, Andreas Fach, Tiara Ratz

**Affiliations:** 1 Health Promotion and Prevention Unit, Department of Health Sciences, Hamburg University of Applied Sciences/Hochschule für Angewandte Wissenschaften Hamburg (HAW Hamburg), Hamburg, Germany; 2 Health Psychology & Behavioral Medicine Lab, School of Business, Social and Decision Sciences, Constructor University, Bremen, Germany; 3 Apprevent GmbH, Bremen, Germany; 4 Klinikum Links der Weser, Bremen, Germany; 5 AO Innovation Translation Center, Clinical Operations, AO Foundation, Switzerland

**Keywords:** cardiovascular diseases (CVDs), non-communicable diseases (NCDs), disease self-management, digital health application, randomized controlled trial, multiple behavior change

## Abstract

**Background:**

Prevention of acute cardiovascular events in patients with cardiovascular disease (CVD) requires promoting health-protective behaviors (e.g., physical activity) and reducing health-compromising behaviors (e.g., sitting). Digital interventions addressing health behavior offer great potential. Based on a multiple behavior change theory, an intervention in the form of a digital health application (app) was evaluated in a pilot trial, testing the following hypotheses (H): H1: Health behaviors (physical activity, sitting) and disease self-management (self-care maintenance, self-care confidence) are closely related; H2: changes in health behaviors and disease self-management indicators over time (T0 to T1) are more pronounced in the intervention group (IG, app users) than in the control group (CG); H3: within the IG, changes in systolic and diastolic blood pressure indicate a positive trajectory.

**Methods:**

A 12-week randomized controlled trial (RCT) was conducted with two measurement points. The IG received an app addressing self-management and health behavior change. A total of *N* = 40 CVD patients were randomized equally to the CG (45% women; mean age = 60.6 years) and the IG (35% women; mean age = 61.5 years).

**Results:**

Findings support H1 with correlations between behaviors (*r* = −0.66–0.79) and disease self-management (*r* = −0.06–0.70). H2 was also partially supported, with significant improvements over time in self-management indicators, especially self-care maintenance, in the IG (Eta² = 0.35; *p* < 0.001). H3 could not be confirmed as no significant changes were found.

**Conclusions:**

This study provides evidence that an app addressing different behavior change techniques (BCTs) can help to manage CVD by promoting health-protective behaviors and preventing health-compromising behaviors. Taking different behaviors into account may increase the effectiveness of behavioral intervention, thereby improving individual and public health. Replications with larger samples and more objective measures are needed.

## 1. Introduction

Public health focuses on protecting and improving the health of individuals and their environments. Promoting healthy lifestyles is key, and while individuals need evidence-based interventions, their environments can also facilitate the integration of such interventions by incorporating technology. In recent years, there has been a growing recognition of priority health problems, emphasizing the importance of promoting health-protective behaviors and preventing health-compromising behaviors (e.g., [1]–[4]). This is because many leading causes of morbidity and mortality, such as cardiovascular disease (CVD), cancer, and diabetes as key non-communicable diseases, are related to modifiable lifestyle behaviors, including physical inactivity and sitting, poor nutrition, and smoking.

On the one hand, physical activity and reduced sitting behaviors can decrease the risk of chronic diseases and other health problems [1],[4] due to improved strength, balance, body composition, cardiorespiratory fitness, flexibility, functional capacity [5], and psychosocial effects [1]. Conversely, engaging in more physical activity and reducing sitting time can help manage the risk of comorbidities from chronic diseases and prevent premature death [4],[6] due to the same physiological and psychological mechanisms. Thus, promoting health-protective behaviors and preventing health-compromising behaviors requires a comprehensive understanding of these behaviors and leveraging technology for support [2],[3].

Regarding the burden of diseases, there are more than 6 million new cases of CVD in the European Union (EU) every year [4]. With almost 49 million people living with CVD, the economic impact in the EU is substantial, totaling €210 billion a year. CVD is the leading cause of death in Europe, accounting for 2.2 million deaths in females and 1.9 million deaths in males. Ischemic heart disease alone accounts for 38% of CVD-related deaths in female and 44% in male patients [4]. Similarly, in Germany and in 2023, CVD and diseases of the circulatory system are the main causes of death, with approximately 180,106 cases or 18% out of 1.028206 deaths due to ICD-10: I11, I21, I25 and I50 [7].

The prevalence of cardiovascular diagnosis increases with age, with men being affected as early as at 45 years old [8]. Hypertension is the most common concomitant diagnosis in CVD and in heart failure patients and, at the same time, one of the main risk factors [9], which calls for action as it correlates with modifiable lifestyle behaviors. However, most myocardial infarction patients fail to change their lifestyle, leaving them at high risk for subsequent clinical events with unnecessary high costs [10],[11]. Medical rehabilitation aims to improve this, though the positive effects of rehabilitation erode over time and cannot sufficiently prevent the reoccurrence of medical conditions [6],[12]–[14]. While special treatments such as the so-called disease management programs (DMPs [15]), particularly for individuals with CHD, have been demonstrated to be effective, it is key to include “close monitoring of patients by the clinicians, along with patient self-management” [16].

In light of the current personnel and budget shortage, as well as difficulties in providing needed patient support, technology has become one significant component of aftercare treatment. This is especially relevant in Germany, where patient behavior indicates significant shortcomings. High rates of inadequate physical activity, insufficient efforts to lower blood pressure, low smoking cessation rates, and poor adherence to guideline-based statin therapy underscore the need for action [15],[17]. Furthermore, compared to other Western European EU countries, Germany continues to fall behind in terms of life expectancy. Accordingly, there is a need to increase the prevention and early detection of CVD [18]. Employing technological solutions is a promising approach, but questions regarding the concrete procedure or specific strategies to efficiently utilize technology remain.

Reducing the risk profile for cardiovascular co-morbidity and mortality is a lifelong task that requires individuals to develop strong self-management abilities [6]. This is also the focus of public health efforts, particularly public health as cardiac secondary and tertiary prevention, which are guided by evidence-based health education and health literacy training [19]. Strengthening health literacy also implies increased self-efficacy [20],[21]. The theoretical construct of self-efficacy (also known as *self-care confidence*; [10]) is a key determinant of health behaviors and correlates with health literacy [22]–[24]; individuals are health literate if they have the confidence and willingness to apply health information to themselves and convert this knowledge into action [21]. Furthermore, ensuring reliable lifestyle-related behaviors, such as avoiding smoking, drinking alcohol in moderation, engaging in regular physical activity, and maintaining a healthy diet, are important [25].

Earlier approaches to implementing mobile rehabilitation have demonstrated that eHealth, mHealth, or dHealth are evidence-based and effective [22]. For example, pilot data from an observational study showed improvement in patients' NYHA class (NYHA: New York Heart Association Functional Classification, an indicator for heart failure severity) after three months compared to baseline. Additionally, patients reported increased disease-related quality of life, self-care, and health literacy [26]. Syntheses of randomized controlled trials (RCTs) further support these findings, reporting successful improvement of blood pressure control through enhanced self-management, also known as *self-care maintenance* [26], in patients with hypertension [27], and a significant reduction in mortality risks in individuals with coronary heart disease [28].

As adherence is low for secondary prevention of cardiovascular diseases [29], strategies to improve adherence to therapy guidelines are urgently needed, including the incorporation of technological behavioral interventions such as dHealth. However, there is comparatively little evidence for its effectiveness in Germany. To address this gap, Germany passed a policy and subsequent legislation [Digitale Versorgung Gesetz (DVG) and Digitale-Gesundheitsanwendungen-Verordnung (DiGAV)] in 2020. It allows mobile health (mHealth) applications (apps) for the treatment of diseases to be prescribed by physicians and to be fully reimbursed by health insurance, known as so-called *digital health applications (DiGA)* or *dHealth applications*.

The requirements for the DiGA are correspondingly stringent, including a comprehensive certified procedure that requires scientific proof of effectiveness through a comparative study. The basis of a DiGA is a CE marking which stands for “Conformité Européenne” and translates from French to English as “European Conformity”. CE marking states that a product meets the requirements of all applicable EU directives, see https://europa.eu/youreurope/business/product-requirements/labels-markings/ce-marking/index_en.htm. Other requirements include security, functionality, interoperability, data protection, and data security, which clearly differentiates DiGA from other mHealth applications. The DiGA certification process is called a fast-track process, and the evaluation is the responsibility of a government agency (Bundesinstitut für Arzneimittel und Medizinprodukte, BfArM).

MHealth and dHealth applications hold the potential to support patients with CVD in their daily therapy routines while being cost-effective and providing coverage for best practice measures [30],[31]. International studies evaluating apps' effects on monitoring and strengthening the adherence of people with CVD indicate significant improvements in medication adherence [32] and reduced systolic blood pressure [33].

**Figure 1.**
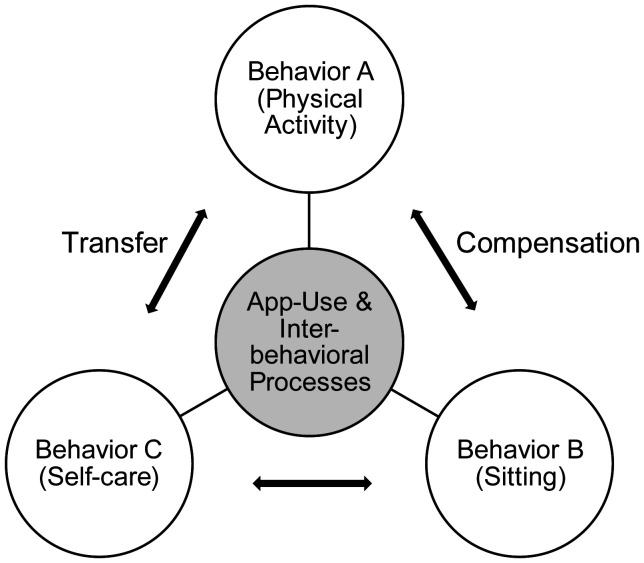
The compensatory carry-over action model (CCAM) applied to app users.

A public health perspective can help to improve such interventions in healthcare or in addition to the clinical healthcare system [19]. The effectiveness of behavior-change interventions across multiple life domains can be elucidated by theories like the compensatory carry-over action model (CCAM) (Figure 1) [30]. The CCAM explains how behavior changes in one domain can transfer into another behavior or, alternatively, lead to compensatory behaviors in a different domain. Concretely, self-care and physical activity are behaviors that the CCAM predicts will correlate positively with each other while correlating negatively with sitting [34]. The model has important implications for understanding how behavior change interventions can be designed [35] to promote sustainable behavior change across multiple domains of life. Because little is known about the interrelations of different behaviors over time and with each other, the CCAM proposes psychological mechanisms between the different behaviors, such as transfer between domains [34].

While there is consensus that technology provides significant potential for innovations in healthcare [2], so far, few clinical randomized studies of apps certified as medical device software have been published, particularly those tested in the fast-track process (DiGA) and aimed at strengthening adherence in CVD patients in Germany.

The reCardial app, which met all technical requirements for a DiGA application, was initially used for provisional listing. Therefore, the aim of this study was to explain the intervention approach from a public health and behavioral psychology perspective and to explore the interrelations between behaviors and the effects of the reCardial app on disease self-management and health behavior change in patients with hypertension, as well as concomitant chronic ischemic heart disease and/or heart failure within the design of an RCT. The following hypotheses (H) will be tested:

H1: Health behaviors (total physical activity and sitting) and disease self-management indicators (self-care maintenance, self-care confidence) are closely interrelated.

H2: Changes in health behaviors and disease self-management indicators over time (T0–T1) are more pronounced in the intervention group (IG, app-users) than in the control group (CG).

H3: In the IG, changes in systolic and diastolic blood pressure indicate a positive trajectory.

## 2. Materials and methods

The study was conducted in accordance with the Declaration of Helsinki and approved by the Institutional Review Board (or Ethics Committee) of Ethikkommission der Ärztekammer Bremen (protocol code with the ethical approval code 762 October 15, 2021). The randomized controlled trial (RCT) has been prospectively registered in the German Clinical Trials Registry (identification number: DRKS00026136). The study had the title reCardial—Smartphone-Applikation zur Stärkung der Therapieleitlinien-Adhärenz bei Patienten mit Hypertonie, chronischen ischämischen Herzkrankheiten oder Herzinsuffizienz” [reCardial - smartphone application to strengthen therapy guideline adherence in patients with hypertension, chronic ischemic heart disease or heart failure].

### 2.1. Design

**Figure 2.**
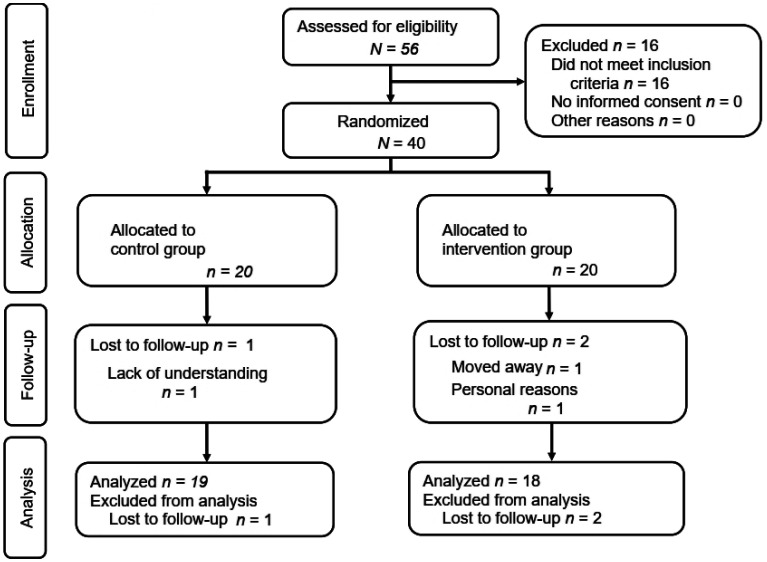
Flow diagram of study design.

A randomized controlled and superiority trial with a pilot study methodology was conducted within the German medical context and public health setting, i.e., where behavioral effects, in addition to physiological effects, were taken into account. Eligible patients (*N* = 40) were randomly assigned to one of two parallel groups: the waiting list CG (*n* = 20) or the IG (*n* = 20) using a 1:1 allocation (see Figure 2).

The intervention group (IG) received access to the reCardial app in addition to standard care for 12 weeks. The behavioral intervention, in the form of access to an app supporting the self-management of CVD, was provided in the patient's daily care routines at home. To accurately portray the German standard healthcare system, the control group (CG) received only “care as usual” (without access to the app). Care as usual is defined as evidence-based standard care in accordance with current therapy guidelines or as realized in disease management programs [9],[12],[14]–[16]. This approach provided information for general practice care in accordance with the guidelines. Control visits usually occurred on a quarterly basis. After the 12-week waiting period and the follow-up assessment, the CG was granted access to the app.

### 2.2. Recruitment and eligibility criteria

Study participants were recruited by general practitioners and specialists in one city-state region. The general practitioners and specialists distributed flyers directing their patients to the study website. Furthermore, recruitment took place through cardiac sports groups, social media calls, and press releases.

The inclusion criteria required a diagnosis of essential (primary) hypertension (ICD-10: I10). Individuals with concomitant chronic ischemic heart disease (ICD-10: I25) and/or mild to moderate heart failure (ICD-10: I50; NYHA, functional classification of stages I–III) were also eligible. Further inclusion criteria included the patient's informed consent, being at least 18 years of age, the ability to read and understand German, regularly taking antihypertensive medication, and owning and being able to use a smartphone.

Patients were excluded from the study (*n* = 16; see Figure 2) if any of the following applied: having experienced an acute cardiovascular event (e.g., a myocardial infarction) within the previous 4 weeks, pregnancy, active psychosis, severe dementia (in accordance with the app's instructions for use), prior use of the app, or simultaneous participation in another research study. Additional contraindications for app usage were defined and led to the exclusion from the study (see Table S1 in Supplementary Information).

### 2.3. Informed consent and participant flow

The study website provided potential study participants with comprehensive information about the study, as well as an email address and telephone number to contact the study team for questions. The study information, data security statement, and informed consent form were available for download on the study website.

After reviewing the study information on the survey software, individuals were asked to confirm that none of the exclusion criteria applied and that they met the inclusion criteria by checking the respective boxes on the study website. Furthermore, they were informed that, by checking another box, they confirmed their informed consent (all patients assessed for eligibility provided their informed consent). By confirming the requirements and clicking “participate”, individuals were included in the study and subsequently directed to the online baseline questionnaire. By means of a phone call, verification of eligibility criteria was checked and only eligible patients were retained in the study (which was the case of all patients assessed for eligibility).

Subsequently, individuals received an email sent to the email address they provided when completing the baseline questionnaire, informing them of their access to the app instructions or care as usual only (which also disclosed the random group allocation to the patients). Randomization was realized using the survey tool's “random trigger” function, which randomly assigned the study participants to either the CG or IG.

Participants assigned to the IG received an email containing the download link and QR code for the app. To link the information in the online questionnaire with the app data, participants were asked to generate their personal identification code in the online questionnaire. The concept of a self-generated identification code (SGIC) was used, with code elements based on the individual person [36]. Study participants in the IG were also required to enter this identification code in a designated field in the app.

Study participation could be canceled at any time upon request by the participants. No criteria for deterioration of the medical condition were pre-defined, as this was monitored during the study. No intervention modifications were planned or performed throughout this study. As an incentive, all study participants received a blood pressure device with instructions to regularly measure their blood pressure. This device could be kept at the end of the study. Participants also received a small financial reward under the condition they completed the 12-week follow-up online questionnaire. They were required to refrain from undergoing alternative treatment options that required simultaneous participation in another study. Patients were advised to consult their physician before pursuing any concomitant care not defined as standard care or as part of a certified disease management program (to qualify as care as usual).

### 2.4. Behavioral intervention

In this RCT, the reCardial app, in addition to standard care, was defined as the intervention. It was developed by a multidisciplinary team of experts in medical practice and public health research, integrating stakeholders' needs through a co-design strategy [37]. The app was designed in accordance with the currently valid therapy guidelines [16], applied evidence-based theories [34],[38], and utilized behavior change techniques [35],[39] to support patients in the everyday management of their condition [27],[40],[41]. As essential hypertension, heart failure, and chronic ischemic heart disease are lifelong conditions and highly dependent on lifestyle, a high level of health literacy and self-management skills is required [16],[20],[21]. Patients must continuously monitor and plan relevant parameters, such as regular medication intake, physical activity, and measurements of blood pressure and/or body weight. Thus, the app was designed to complement standard care by enhancing self-management skills and strengthening adherence to therapy guidelines.

The app was a self-management tool for the daily monitoring and prevention of cardiovascular risk factors. It aimed to support the implementation of and adherence to therapy guidelines for the treatment and secondary/tertiary prevention of CVD (see Table 1). The app included the following main features: a reminder function for scheduled medications, training, and vital data (such as blood pressure) measurements, self-monitoring of recommended health behaviors and health measurements, and health and self-management education, all of which have been demonstrated to be effective [22],[40],[42]–[44]. The app was available for iOS and Android. The intervention content was grounded in psychological theories and behavior change strategies (BCTs) [34],[35],[38] designed to help promote relevant predictors of behavior change by initiating so-called mechanisms of action [39]. The BCTs addressed in the app are outlined in Table 1.

**Table 1.** Intervention content and addressed behavior change techniques (BCTs [35]).

*BCT number*	*Formal description*	*Concrete content for patients*
1.1	Goal setting (behavior)	Select relevant modules to focus on (steps, medication, and training) Encouragement to set goals
1.2	Problem-solving/coping planning	Identify barriers to health behavior change and generation of if-then plans
1.3	Goal setting (outcome)	Quizzes to test their health knowledge
1.4	Action planning	Training in planning and implementation intentions
1.5	Review behavior goal(s)	Select relevant modules to focus on (steps, medication, and training) Encouragement to set goals
1.6	Discrepancy between current behavior and goal standard	Tracking of adherence to behavioral goals regarding planned medications and training
1.7	Review outcome goal(s)	Surveys with the aim to increase to evaluate health behavior
1.8	Behavioral contract	Targets defined by patients and physicians
1.9	Commitment	Targets defined by patients and physicians
2.2	Feedback on behavior	Under progress, development of daily steps and vital data was graphically displayed
2.3	Self-monitoring of behavior	Documentation/synchronizing data and activities (activity trackers, smart watches, blood pressure monitors, etc.)
2.4	Self-monitoring of the outcome of behavior	Rating of satisfaction with quality of life
2.6	Biofeedback	Biofeedback (display of vital signs)
3.1	Social support (general)	Seeking social support
3.2	Social support (practical)	Mobilizing social support
4.1	Instructions on how to perform a behavior	In consultation with the physician, a medication plan was determined
4.2	Antecedents	Theory-based information and recommendations (texts & videos tailored to the duration of use; see, e.g., [35],[45], structured health education with the aim to increase knowledge)
4.3	Reattribution	Theory-based information and recommendations (texts & videos tailored to the duration of use; see, e.g., [35],[45], structured health education with the aim to increase knowledge)
4.4	Behavioral experiments	Select relevant modules to focus on (steps, medication, and training)
5.1	Information about health consequences	Explanation of relevant cardiovascular risk factors, risk behaviors, and health consequences
5.2	Salience of consequences	Emphasizing the importance of consequences
5.3	Social and environmental consequences	Highlighting health consequences, and praise
5.4	Monitoring of emotional consequences	Rating of quality of life
5.6	Information about emotional consequence	Gamification with streaks for continuous monitoring and meeting of targets and collecting “heart points” for using and adhering to app features
6.1	Demonstration of the behavior	In consultation with the physician, target values for exercise and training were set
6.3	Information about others' approval	In consultation with the physician, weight and blood pressure were defined
7.1	Prompts/cues	Reminders like push notifications with the aim to increase additional support for taking medication, adhering to planned physical activities and blood pressure measurements
7.8	Classical conditioning/associative learning	Push notifications
8.1	Behavioral rehearsal/practice	The monitoring implemented in the app
8.3	Habit formation	Implementing methods with the aim to increase habit formation and maintenance
8.4	Habit reversal	Implementing methods with the aim to increase habit formation and maintenance
8.6	Generalization of a target behavior	Instructions to internalize and implement what had been learned and to transfer it
8.7	Graded tasks	Beginning of each week: “task of the week”
9.1	Persuasive argument	Regular information with advice preference-based
9.2	Pros and cons	Regular information with advice preference-based
9.3	Comparative imaging of future outcomes	Regular information with advice preference-based
10.1	Material incentive (behavior)	App itself worked as such
10.2	Material reward	Trophies or medals
10.3	Nonspecific reward	Different features such as reminders
10.4	Social reward	Positive feedback or recognition from others
10.6	Nonspecific incentive	Critical assessment of progress
10.7	Self-incentive	Critical assessment of progress
10.9	Self-reward	Reminders to appreciate oneself
11.1	Pharmacological support	Select relevant modules to focus on (steps, medication, and training)
11.2	Regulate negative emotions	Trying out a stress management method
11.3	Conserving mental resources	Encouragement to implement behavior change strategies such as setting goals
12.1	Restructuring physical environment	Restructuring environment
12.2	Restructuring social environment	“Locations” module displayed rehabilitation clinics, hospitals with diagnostic and chest pain unit (CPU) & nearby supervised cardiac support groups [41]
12.3	Avoidance/changing exposure to cues for behavior	“Locations” module displayed rehabilitation clinics, hospitals with diagnostic and chest pain unit (CPU) & nearby supervised cardiac support groups [41]
12.5	Adding objects to the environment	Restructuring environment
13.1	Identification of self as a role model	Self-management skills
13.2	Framing/reframing	Self-management skills
13.3	Cognitive dissonance	Health, stress & sleep, social-cognitive determinants of promoting health-protective preventive behaviors
13.4	Valued self-identity/self-affirmation	Regular monitoring, health, stress & sleep, social-cognitive determinants of promoting health-protective preventive behaviors
13.5	Identity associated with changed behavior	Health, stress & sleep, social-cognitive determinants of promoting health-protective preventive behaviors
15.1	Verbal persuasion to boast self-efficacy	Information on self-efficacy (self-care confidence) for maintaining the newly adopted healthy lifestyle long-term
15.2	Mental rehearsal of successful performance	Advice on therapy guideline adherence with the aim to increase intention to change and perceived self-efficacy
15.3	Focus on past success	Visual feedback
15.4	Self-talk	Advice on therapy guideline adherence with the aim to increase intention to change and perceived self-efficacy
16.3	Vicarious reinforcement	Instructions to synchronize results of a blood pressure monitor with app

Additionally, the app collected various indicators of usage and adherence. These included monitoring of blood pressure (average number of synchronized blood pressure readings per week, average systolic and diastolic blood pressure per week), number and percentage of medications marked as taken versus not taken per week, number and percentage of planned workouts marked as completed versus not completed per week, average daily steps per week, number of articles read per week in the “knowledge” module, and number of correct and incorrect answers to weekly quiz questions. Users received push notifications if any adherence indicator had not been logged for seven consecutive days. A further strategy to improve adherence was the implementation of gamification (see Table 1). All instructions and feedback within the app were designed to be as individualized as possible (BCT Tailored personalized message/BCT not in Taxonomy v1).

### 2.5. Outcomes

The following parameters were investigated as endpoints:

- Change in adherence to therapy guidelines after 12 weeks, operationalized using the Self-Care of Hypertension Inventory (SC-HI) score, section A (self-care maintenance), Version 2.0 [46]. This section consisted of eleven items covering recommended self-care activities, measured on a four-point Likert scale. The SC-HI was analyzed using the standardized score, with values potentially ranging from 0–100 (higher values indicating higher self-care). A difference of eight points and a standard deviation (*SD*) of 16 was considered clinically relevant [47],[48].

- Self-care confidence/self-efficacy at baseline and after 12 weeks, measured by the SC-HI score, section C, Version 2.0 [46]. This section consisted of six items, measured on a four-point Likert scale, with standardized scores ranging from 0–100.

- Total physical activity at baseline and after 12 weeks, measured by the short version of the International Physical Activity Questionnaire Short Form (IPAQ-S). The short version contained seven items with open-ended questions about physical activities and sedentary behaviors over the past seven days [49]. The total minutes spent walking (at least ten minutes per week) and performing moderate and vigorous activities per week were multiplied by the metabolic equivalent of task (MET) to calculate the total physical activity in MET minutes per week. Sitting behavior was assessed separately, based on hours spent sitting per day. The IPAQ-S was applied not only to healthy populations but also to those suffering from chronic diseases [50]. Previous studies using the IPAQ included patients diagnosed with hypertension, coronary heart disease, and/or heart failure, using apps as models [51]. A difference of 200 MET minutes (*SD* = 300) is considered clinically relevant [52].

- Self-measured systolic and diastolic blood pressure in mmHg at baseline and after 12 weeks within the IG, using the Omron M400 Intelli IT, a calibrated blood pressure monitor (sphygmomanometer, see e.g., [53]).

Additional demographic information was collected at baseline. The baseline questionnaire recorded sex, date of birth, and the highest level of education. Participants were also asked whether they had any comorbidities (i.e., chronic ischemic heart disease and/or heart failure).

### 2.6. Statistical analyses

To test H1, correlation analyses were performed (Spearman). To test H2, ANCOVAs were conducted to assess the superiority of the intervention over the control condition, adjusted for baseline values, sex, and age. Further, *t*-tests were performed within the descriptive analyses. To test H3, paired *t*-tests were used to analyze mean differences in systolic and diastolic blood pressure in mmHg.

For testing H2 and H3, the precision of effect sizes was ensured with a 95% confidence interval (95% *CI*), and a two-sided *p*-value < 0.05 was considered statistically significant. Since this was a pilot study in terms of methodology, no alpha adjustment was made. The difference in differences (DID) was calculated as the difference between baseline and follow-up in the group comparison. In addition, as this was a pilot study and no power calculation was performed, a minimum sample size of *n* = 40 was set based on previous research [54]. Statistical analyses were performed with R statistical software (version 4.2.2.).

## 3. Results

### 3.1. Study population characteristics

In total, *n* = 37 (92.5%) participants completed this study (Figure 2). The characteristics of the study population at baseline are presented in Table 2. A total of 25% (CG) and 30% (IG) of the study participants were ≥70 years old at baseline. The median age was 61.0 years (interquartile range, IQR: 55.0–69.8) in the CG and 63.0 years (IQR: 56.0–70.8) in the IG. The proportion of female participants was 45% in the CG and 35% in the IG. In the CG, 45% had another concomitant CVD, while in the IG, 70% were comorbid (Table 2).

**Table 2.** Sociodemographic and clinical characteristics of the study population at baseline by randomization.

Project	Control group (CG) *n* = 20 *M* (*SD*) or *n* (%)	Intervention group (IG) *n* = 20 *M* (*SD*) or *n* (%)
**Age in years**	60.6 (10.6)	61.5 (13.4)
**Age in categories (1)**		
≤50 years	3 (15.0%)	3 (15.0%)
51–60 years	7 (35.0%)	4 (20.0%)
61–70 years	6 (30.0%)	8 (40.0%)
>70 years	4 (20.0%)	5 (25.0%)
**Age in categories (2)**		
<70 years	15 (75.0%)	14 (70.0%)
≥70 years	5 (25.0%)	6 (30.0%)
**Sex**		
Female	9 (45.0%)	7 (35.0%)
Male	11 (55.0%)	13 (65.0%)
Diverse	0	0
Education (highest school-leaving qualification*)		
Low	5 (25.0%)	6 (30.0%)
Middle	7 (35.0%)	7 (35.0%)
High	8 (40.0%)	7 (35.0%)
**Comorbidity**		
Chronic ischemic heart disease (ICD-10: I25)	6 (30.0%)	10 (50.0%)
Heart failure (ICD-10: I50; NYHA I–III)	3 (15.0%)	4 (20.0%)

*Note*: *Classification of highest school-leaving qualification: low = 9 years of schooling (Haupt-/Volkshochschule); middle = 10+ years of schooling (Realschulabschluss/Mittlere Reife/Fachschulreife); high = 12 years of schooling (Abitur/Allgemeine or fachgebundene Hochschulreife/erweiterte Oberschule); *M* = mean; *SD* = standard deviation.

### 3.2. Interrelation of health behaviors and disease self-management indicators

To test H1 (i.e., that health behaviors and disease self-management indicators are closely interrelated), correlation analyses were conducted. Findings support H1 with significant correlations observed between behaviors (*r* = −0.66–0.79) and with disease self-management (*r* = −0.06–0.70). Further, the results in Table 3 indicate that cross-sectionally (only at T0), self-care maintenance, and confidence were significantly correlated with physical activity (*r* = 0.34–0.59). The same pattern appeared at T1 (*r* = 0.27–0.79).

In detail, sitting was negatively correlated with physical activity, such that the patients with more sitting time at T0 were less likely to be physically active at T1 (*r* = −0.48), and those sitting more at T1 were also less physically active at T1 (*r* = −0.54), with the IG revealing even higher correlations than the CG (Table 3).

The highest correlations between health behaviors and disease self-management indicators emerged cross-sectionally (within T0 or within T1) between self-care maintenance and physical activity (*r*_T0_ = 0.59 and *r*_T1_ = 0.44, higher correlation in the IG), as well as between self-care confidence and physical activity (*r*_T0_ = 0.46 and *r*_T1_ = 0.52, also higher correlation in the IG).

Focusing only on the longitudinal (T0–T1) interrelations, there was a strong correlation between self-care maintenance at T1 and physical activity at T0 (*r* = 0.58), in contrast to the nonsignificant correlation between self-care maintenance at T0 and physical activity at T1 (*r* = 0.08). Similarly, as previously reported, there was a negative correlation between sitting at T0 and physical activity at T1 (*r* = −0.48, Table 3).

The results regarding H1 can be summarized as follows: Health behaviors, including sitting and physical activity, and disease self-management indicators such as self-care maintenance and self-care confidence, were interrelated, providing support for H1. Specifically, self-care maintenance and physical activity showed shared variance; self-care confidence and physical activity were linked cross-sectionally; and sitting time and physical activity were inversely associated.

### 3.3. Change in health behaviors and disease self-management indicators between groups over time

To test H2 (i.e., that change in health behaviors and disease self-management indicators from T0–T1 is more pronounced in the IG compared to the CG), ANCOVAs adjusted for baseline score, sex, and age were conducted. The results showed significant differences between the groups for self-care maintenance (*p* < 0.001) with Eta² = 0.42 (see Table 4) and self-care confidence (*p* = 0.04) with Eta² = 0.13 (Table 5). The effect sizes fall within the small and medium range, indicating clinical importance [47],[48].

No other dependent variables revealed significant effects (see Supplementary Information, Tables S2–S3). No significant differences were found between the groups at baseline. At T1, the mean values in the IG for self-care maintenance improved by 18 points (*SD* = 24.2) compared to −3 points (*SD* = 10.4) in the CG (*p* < 0.001), corresponding to a DID of 21 (*p =* 0.002), which is clinically relevant or considerably higher than the minimal clinically important difference (MCID) [47],[48].

Self-care confidence improved by 6.8 points (*SD* = 25) compared to a decrease of 8.8 points (*SD* = 29.7) in the CG (*p* = 0.046), corresponding to a DID of 15.6 (*p* = 0.092). Thus, in contrast to the IG, the CG showed a slight deterioration in self-management indicators (see Table 6).

**Figure 3.**
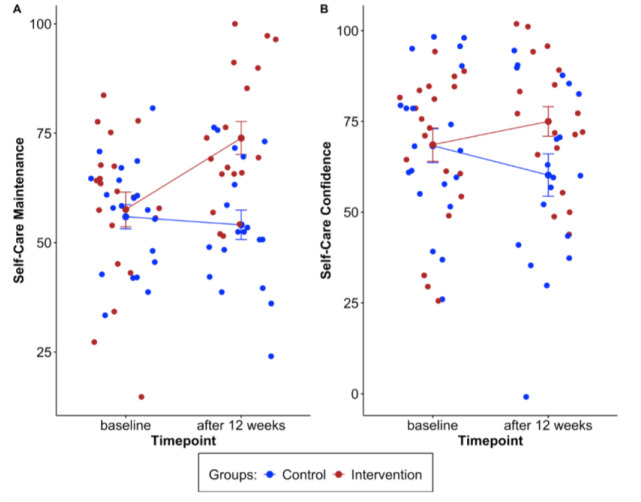
Means with standard error (*SE*) over time (T0–T1) for self-care maintenance (panel A) and confidence (panel B) differentiated by group: control (CG) and intervention (IG).

Figure 3 shows the increase in self-management indicators from T0/baseline to T1/after 3 months in the IG.

**Table 3.** Spearman correlation analyses with correlation coefficients (*r*) for the total sample and, in brackets, for the control group (CG, first coefficient in the brackets) and the intervention group (IG, second coefficient in the brackets).

**Project**	**1**	**2**	**3**	**4**	**5**	**6**	**7**
**1.Self-care maint. T0**							
**2. Self-care maint. T1**	0.40* (0.75**/0.19)						
**3. Self-care conf. T0**	0.45** (0.34/0.55*)	0.10 (0.15/0.17)					
**4. Self-care conf. T1**	0.26 (0.58**/−0.06)	0.63**** (0.57*/0.70**)	0.22 (0.16/0.33)				
**5. Sitting T0**	0.08 (0.15/−0.03)	−0.26 (−0.10/−0.43)	0.33 (0.28/0.35)	−0.30 (−0.13/−0.49)			
**6. Sitting T1**	0.14 (0.04/0.22)	−0.26 (0.22/−0.36)	0.41* (0.33/0.41)	−0.20 (−0.01/−0.34)	0.79**** (0.82**/0.76**)		
**7. Phys. activity T0**	0.59** (0.46/0.69*)	0.58** (0.48/0.58)	0.46* (0.34/0.53)	0.31 (0.14/0.27)	−0.33 (−0.30/−0.23)	−0.44 (−0.47/−0.65)	
**8. Phys. activity T1**	0.08 (0.30/−0.22)	0.44* (0.27/0.78*)	−0.01 (0.16/−0.02)	0.52** (0.42/0.79**)	−0.48* (−0.41/−0.63)	−0.54** (−0.45/−0.62*)	0.64** (0.66*/0.36)

Note: ****p < 0.0001; ***p < 0.001; **p < 0.01; *p < 0.05.

**Table 4.** Results of the ANCOVA with self-care maintenance as the dependent variable, adjusted for the baseline score (T0), sex, and age.

**Project**	***Sum Sq***	***df***	***F value***	***p* (>*F*)**	**Effect size**	
					**Eta^2^ partial**	**95% *CI***
(Intercept)	15.28	1	0.08	0.77		
Group	4211	1	23.2	<0.001***	0.42	[0.16, 0.61]
Self-care T0	4140	1	22.81	<0.001***	0.42	[0.16, 0.61]
Sex	254.3	1	1.40	0.25	0.04	[0.00, 0.24]
Age	1841	1	10.14	0.003**	0.24	[0.03, 0.46]
Residuals	5807	32				

Note: ***p < 0.001; **p < 0.01; *p < 0.05.

**Table 5.** Results of the ANCOVA with self-care confidence/self-efficacy as the dependent variable, adjusted for the baseline score (T0), sex, and age.

**Project**	***Sum Sq***	***df***	***F value***	***p* (>*F*)**	**Effect size**	
					**Eta^2^ partial**	**95% *CI***
(Intercept)	294.1	1	0.62	0.44		
Group	2248	1	4.71	0.04*	0.13	[0.00, 0.35]
Self-Eff. T0	9385	1	19.68	<0.001***	0.38	[0.13, 0.58]
Sex	52.17	1	0.11	0.74	0.003	[0.00, 0.13]
Age	862.3	1	1.81	0.19	0.05	[0.00, 0.26]
Residuals	15,261	32				

Note: ***p < 0.001; **p < 0.01; *p < 0.05.

Regarding the evaluation of the IPAQ-S, descriptive differences did not relate to significant differences, neither for total physical activity nor for the individual components of physical activity (walking, moderate, and vigorous activities) or sitting (see Table 6).

**Table 6.** Means (*M*), standard deviations (*SD*), and difference in difference (DID) of the health behaviors and disease self-management indicators at baseline (T0) and after 12 weeks (T1).

**Project**	**Control group (CG) *M* (*SD*)**			**Intervention group (IG) *M* (*SD*)**			**Between groups, *p*-value**		**DID, *p*-value**

	**T0**	**T1**	**T1–T0**	**T0**	**T1**	**T1–T0**	**T0**	**T1**	
**Self-care maintenance**	55.9 (12.3); *n* = 20	54.1 (14.6); *n* = 19	−3.0 (10.4); *n* = 19	57.6 (17.7); *n* = 20	73.9 (16.0); *n* = 18	18.0 (24.2); *n* = 18	*p* = 0.732	*p* < 0.001	21.0; *p* = 0.002
**Self-care confidence**	68.3 (20.9); *n* = 20	60.2 (25.5); *n* = 19	−8.8 (29.7); *N* = 19	68.6 (20.5); *n* = 20	75.0 (17.3); *n* = 18	6.8 (25.0); *n* = 18	*p* = 0.966	*p* = 0.046	15.6; *p* = 0.092
**Total physical activity**	3809.1 (3796.9); *n* = 14	3843.4 (3656.7); *n* = 17	−604.7 (2311.9); *n* = 13	5326.9 (4538.4); *n* = 11	5151.5 (2887.2); *n* = 13	−1640.1 (4842.6); *n* = 7	*p* = 0.384	*p* = 0.283	1035.5; *p* = 0.609
Vigorous activity	1422.9 (1629.0); *n* = 14	2054.1 (2520.0); *n* = 17	73.9 (1684.0); *n* = 17	2160.0 (2562.5); *n* = 11	2215.4 (1466.9); *n* = 13	754.3 (3176.2); *n* = 7	*p* = 0.418	*p* = 0.828	828.1; *p* = 0.539
Moderate activity	1182.9 (1211.8); *n* = 14	889.4 (1200.8); *n* = 17	−360.0 (832.8); *n* = 13	1236.4 (1586.9); *n* = 11	1473.9 (1169.7); *n* = 13	468.6 (1776.0); *n* = 7	*p* = 0.927	*p* = 0.192	108.6; *p* = 0.883
Walking activity	1203.4 (1448.8); *n* = 14	899.8 (1080.7); *n* = 17	−318.5 (737.6); *n* = 13	1930.6 (1102.7); *n* = 11	1462.2 (774.2); *n* = 13	−417.3 (737.5); *n* = 7	*p* = 0.168	*p* = 0.108	98.8; *p* = 0.780
**Sitting**	6.9 (3.4); *n* = 17	6.3 (3.2); *n* = 15	−0.9 (1.6); *n* = 15	5.7 (2.4); *n* = 15	5.6 (2.4); *n* = 14	−0.1 (1.3); *n* = 12	*p* = 0.223	*p* = 0.475	0.8; *p* = 0.174

*Note*: *M* = mean; *SD* = standard deviation; DID = difference in differences.

Regarding H2 (i.e., that the change in health behaviors and disease self-management indicators over time from T0–T1 was more pronounced in the IG in comparison to the CG), to conclude, support was found as expected but only for self-management indicators at a statistical level [47],[48]. Just focusing on the IG, no statistically significant difference was found below the clinically relevant difference of 200 MET minutes [52], but descriptive improvements for vigorous activity (55.4 subtracting the mean scores in Table 6, or 754.3 T1–T0 score in Table 6) and moderate activity (237.5 subtracting the mean scores in Table 6, or 468.6 T1–T0 score in Table 6) in the IG were found (but not total physical activity as walking decreased over time; see Table 6 T0 and T1 means as well as T1–T0 scores). To summarize, H2 was partially supported, with significant improvements over time in self-management indicators, especially self-care maintenance (Eta² = 0.42; *p* < 0.001, Table 4) and only marginally with self-care confidence (Eta² = 0.13; *p* = 0.04, Table 5), as well as descriptive improvements in physical activity (Table 6).

### 3.4. Changes in systolic and diastolic blood pressure in the intervention group

H3 (i.e., that the IG reveals improvements in systolic and diastolic blood pressure, mmHg) was tested using data from study participants in the IG, who monitored their values via the app during the study. Additional monitored values included daily step counts and weight checks.

Table 7 shows the data of the blood pressure monitoring of the IG at baseline and week 12. The mean systolic blood pressure was *M* = 137 mmHg (*SD* = 15.8 mmHg) at baseline (T0) and *M* = 135.3 mmHg (*SD* = 17.7 mmHg) at week 12 (T1, Table 7). The difference between these two measurement times with *d* = 0.101was not significantly different (*p* = 0.444). The mean diastolic blood pressure was *M* = 80.9 mmHg (*SD* = 10.3 mmHg) at baseline and *M* = 81.8 mmHg (*SD* = 8.7 mmHg) at week 12. The difference of *d* = 0.095was also not significantly different (*p* = 0.695).

**Table 7.** Results regarding systolic and diastolic blood pressure in mmHg at T0 and T1 (after 12 weeks after T0), self-assessed by the study participants and recorded in the app.

**Intervention group**	**T0, *M* (*SD*)**	**T1, *M* (*SD*)**	**Difference within group (T0–T1) *M* (*SD*)**
Systolic blood pressure in mmHg	137.0 (15.8); *n* = 15	135.3 (17.7); *n* = 17	−2.7 (7.9); *n* = *6*; *p* = 0.444
Diastolic blood pressure in mmHg	80.9 (10.3); *n* = 15	81.8 (8.7); *n* = 17	1.3 (7.9); *n* = 6 *p* = 0.695

*Note*: *M* = mean (averaged values over seven days); *SD* = standard deviation.

To conclude our results about H3, only on a descriptive level, evidence was found that the IG improved in the objective measure of systolic blood pressure in mmHg as hypothesized, but not in the diastolic blood pressure in mmHg. However, the difference of 2.7 mmHg for systolic blood pressure did not reach statistical significance (see Table 5).

## 4. Discussion

This theory-based study in the field of public health aimed to understand the interrelations of different behaviors and the effect of using a medical app in CVD patients, specifically to become a DiGA, which would be a special dHealth application. It was found that health behaviors and disease self-management indicators were closely interrelated. However, not all health behaviors and disease self-management indicators were significantly correlated (see Table 3), indicating that behaviors were more likely to drive subsequent self-care confidence and maintenance, while sitting appeared to inhibit subsequent physical activity rather than the other way around. Nonetheless, the correlational nature of these data does not allow causal conclusions, which is why the experimental effects between the IG and the CG were tested. In addition, there is the risk of inflation of Type 1 errors as a few repeated tests were performed without a correction; this is a significant risk given the sample size of this research due to its pilot character.

Changes in disease self-management indicators over time (T0–T1, both maintenance and confidence) were more pronounced in users of the app combined with standard care than in those receiving standard care alone. The clinical difference of 21.0 DID value for self-care maintenance and 15.6 DID value for self-care confidence (Table 6) was higher than described in the literature by Riegel et al. [48], although only the DID value for self-care maintenance was statistically significant. Although the app's effectiveness was only partially demonstrated, the observed effects are imperative because self-management can facilitate further behavior change and health improvements in the long term [47],[48]. The effects reported in this study were larger than those in comparable studies with similarly small sample sizes. However, the low statistical power in the current study likely diminished the statistical significance of the findings.

Scientific evaluations of digital health applications in the area of CVD self-management and adherence to therapy guidelines in particular are scarce and have relatively rarely been reported in the German healthcare context. However, existing evidence does support the assumption that digital health applications can provide valuable and clinically relevant improvements in patient-relevant health indicators [22],[30]–[33],[41],[45],[55]–[57].

In a systematic review [58] on the effectiveness of apps for CVD self-management, only two out of the eight included studies were randomized controlled trials—the gold standard for testing such interventions—which was ensured in the current study. Previous studies have shown that mobile self-management apps can reduce hospital admissions, cholesterol levels, and blood pressure and may enhance disease-specific knowledge as well as psychological well-being [58]. This study adds to the understanding that baseline behavior determines subsequent self-management and physical activity, which the effect appearing even stronger in the intervention group.

Another meta-analysis [59] found beneficial effects of digital health applications on reducing heart failure–related hospitalization and improving quality of life. However, no statistically significant effects were reported on all-cause mortality, cardiovascular mortality, all-cause hospitalization, or self-care. While the present study did not test for all these outcomes, the effects observed regarding self-care maintenance may indicate more significant positive effects than in previous studies [59]. This suggests the effectiveness of the app in driving behavior change and the success of the co-creative development approach [60]. The current study ensured high intervention and measurement quality, while not being able to recruit more patients into such a longitudinal study due to its pilot study nature and limited resources. Accordingly, the current research should serve as a foundation for larger trials with more study participants, extended follow-up measurement points, and more outcome measures.

The worldwide changes due to personnel shortages and the implications on healthcare systems have led to increased attention to digital health technologies [2]. Accordingly, our data adds to the evidence that apps can help with the secondary and tertiary prevention of CVD, as well as public health in general. Also, this study demonstrates the usefulness of a theory-driven approach for supporting the understanding of multiple behavior changes and their relation to physiological outcomes. This further underscores the importance of promoting health-protective behaviors and preventing health-compromising ones [22],[34],[35],[38]–[41],[44],[61].

Targeting modifiable risk factors and promoting behavioral changes to prevent recurrent cardiac events is of high clinical importance [6],[21]. Public health plays a crucial role in making the behavioral effects in the population more observable, thereby impacting physiologically determining factors. Accordingly, recognizing these behavioral effects is of elevated importance. Furthermore, a study has found the complexity of medication regimens to be inversely associated with medication adherence and blood pressure management in individuals diagnosed with hypertension [22],[56],[58],[62].

The advantages of dHealth and mHealth tools, such as the described BCT in the reCardial app—including regular reminders, relevant information accessible anytime, and concise visualization of the medication plan—could provide the guidance needed to improve adherence to recommended health and disease management behaviors. However, while the app targeted different BCTs, we were unable to identify which components were particularly efficient and effective. Thus, it remains open whether specific BCTs were working especially well. Another critical consideration is the inequality of the IG and the CG in terms of comorbidities, despite randomization: the CG seemed to be much more vulnerable, which might have led to greater resistance to change and a reduced ability to benefit from the intervention. This calls for further investigations replicating this theory-based, experimental study in the field of public health, including more objective data with larger sample sizes, and extended follow-up measurement points.

Future research should also evaluate the app's impact on multiple behavior changes since only effects on single behaviors as well as its impacts on other disabilities have been explored so far [5],[20]. Replicating these findings with a larger sample size should also include the evaluation of mechanisms, e.g., if more behavior change also leads to better physiological outcomes. However, due to the pilot nature of this study and the limited number of participants, such analyses were not conducted. Moreover, in the future, closer cooperation with stakeholders in co-creative, participatory health research should ensure the impact of this study on the target group [60].

Extending the view to the digital media field for behavior change is important for both healthcare providers and app developers [2]. While evidence for the effectiveness of digital behavioral interventions in changing behavior is limited, this study provides a valuable example of rigorous research, even with a relatively small sample size, particularly with a DiGA approach. However, in the future, actual app-use behavior and interbehavioral processes should be measured to test for their mechanisms. These results are promising in that an app can help individuals improve their disease management and, consequently, their systolic blood pressure at least on a descriptive level.

## 5. Conclusions

This study provides implications for strengthening the evidence-based improvement of public health and healthcare regarding secondary and tertiary prevention of CVD, specifically through the use of a dHealth application. Behavioral interventions should be provided to patients with hypertension, chronic ischemic heart disease, and heart failure via apps to help them manage their lifestyle and health constraints.

Motivating individuals to exhibit a healthy lifestyle, including reduced sedentary behavior and sufficient physical activity, requires health literacy and monitoring skills. These needs can be addressed through the behavior change techniques outlined in this paper and the dHealth applications. Strategies such as setting up plans and defining target values, monitoring, goal setting, enhancing self-efficacy, providing reminders, and delivering knowledge and information about, e.g., nearby supervised cardiac support groups are recommended and should be integrated into dHealth interventions in the future.

## Data availability statement

Data are of a sensitive nature as it includes personal health information. Thus, participants were assured that their raw data remained confidential and would not be shared. The final pseudonymized trial dataset will be made available only from the corresponding author upon reasonable request.

## Use of AI tools declaration

During the preparation of this manuscript, the authors used AI tools in order to improve the readability of certain sentences. After using these tools, the authors reviewed and edited the content as needed and took full responsibility for the content of the publication. The researchers adhere to the EU-AI Act and uphold the basic principles of scientific integrity.


