# Supplementary material for: Health behavior and disease self-management indicators in patients with cardiovascular diseases using a health app: Findings from an RCT
Source: AIMS Public Health. 2025 Feb 26;12(1):233–58. doi: 10.3934/publichealth.2025015 (PMC11999809; doi:10.3934/publichealth.2025015)
Supplement: Supplementary file 1 [file publichealth-12-01-015-s001.pdf]

Public Health

AIMS Public Health, 12(1): 233–258.
DOI: 10.3934/publichealth.2025015
Received: 30 August 2024
Revised: 22 October 2024
Accepted: 08 January 2025
Published: 26 February 2025

https://www.aimspress.com/journal/aimsph

Research article

Health behavior and disease self-management indicators in patients with cardiovascular diseases using a health app: Findings from an RCT

Sonia Lippke[1,2,*], Luisa Korte[3], Vinayak Anand Kumar[2], Andreas Fach[4] and Tiara Ratz[5]

[1] Health Promotion and Prevention Unit, Department of Health Sciences, Hamburg University of Applied Sciences/Hochschule für Angewandte Wissenschaften Hamburg (HAW Hamburg), Hamburg, Germany
[2] Health Psychology & Behavioral Medicine Lab, School of Business, Social and Decision Sciences, Constructor University, Bremen, Germany
[3] Apprevent GmbH, Bremen, Germany
[4] Klinikum Links der Weser, Bremen, Germany
[5] AO Innovation Translation Center, Clinical Operations, AO Foundation, Switzerland

* **Correspondence:** Email: Sonia.Lippke@haw-hamburg.de; Tel: +494212004730.

Supplementary

Table S1. Information on medical contraindications of using the app (exclusion criteria).

ICD-10 Code	Description of ICD-10 Code
A41.0	Sepsis due to Staphylococcus aureus
E05	Thyrotoxicosis [hyperthyroidism]
I24.9	Acute ischemic heart disease, unspecified
I26.0	Pulmonary embolism with acute cor pulmonale
I33	Acute and subacute endocarditis
I40	Acute myocarditis
I50.05	Congestive heart failure with symptoms at rest
I50.14	Left ventricular failure: With symptoms at rest
I71	Aortic aneurysm and dissection
I80	Phlebitis and thrombophlebitis
Z73	Problems related to life-management difficulty

Table S2. Results of the ANCOVA with sitting (in HRS per day) as dependent variable, adjusted for baseline (T0), sex and age.

**Project**	***Sum Sq***	***df***	***F value***	***p* (>*F*)**	**Effect Size**	
					**Eta^2 partial**	**95% *CI***
(Intercept)	3.36	1	1.71	0.21		
Group	0.00	1	0.00	0.96	0.00	[0.00, 0.00]
SittingT_0	10.86	1	5.53	0.03*	0.20	[0.00, 0.47]
Sex	4.26	1	2.17	0.16	0.09	[0.00, 0.35]
Age	3.53	1	1.8	0.19	0.08	[0.00, 0.33]
Residuals	43.19	22				

Note: ***p < 0.001; **p < 0.01; *p < 0.05.

Table S3. Results of the ANCOVA with physical activity (total MET in min per week) as dependent variable, adjusted for baseline (T0), sex and age.

**Project**	***Sum Sq***	***df***	***F value***	***p* (>*F*)**	**Effect Size**	
					**Eta^2 partial**	**95% *CI***
(Intercept)	9,012,174	1	1.90	0.19		
Group	782,502	1	0.17	0.69	0.01	[0.00, 0.26]
Physical ActivityT_0	89,125,109	1	18.74	<0.001***	0.56	[0.18, 0.75]
Sex	7,116,081	1	1.5	0.24	0.09	[0.00, 0.41]
Age	449,493	1	0.10	0.76	0.00	[0.00, 0.24]
Residuals	71,344,851	15				

Note: ***p < 0.001; **p < 0.01; *p < 0.05.

AIMS Press

© 2025 the Author(s), licensee AIMS Press. This is an open access article distributed under the terms of the Creative Commons Attribution License (https://creativecommons.org/licenses/by/4.0)
